# Awareness of predatory journals and open access publishing among orthopaedic and trauma surgeons – results from an online survey in Germany

**DOI:** 10.1186/s12891-021-04223-7

**Published:** 2021-04-17

**Authors:** Elke Maurer, Nike Walter, Tina Histing, Lydia Anastasopoulou, Thaqif El Khassawna, Lisa Wenzel, Volker Alt, Markus Rupp

**Affiliations:** 1 Department of Trauma and Reconstructive Surgery, BG Unfallklinik Tuebingen, Eberhard Karls University Tuebingen, Tuebingen, Germany; 2 Department of Trauma Surgery, University Medical Center Regensburg, Regensburg, Germany; 3 Department of Trauma, Hand and Reconstructive Surgery, University Hospital Giessen, Giessen, Germany; 4 Experimental Trauma Surgery, Justus-Liebig-University Giessen, Giessen, Germany; 5 Faculty of Health Sciences, University of Applied Sciences, Giessen, Germany; 6 Department of Trauma and Reconstructive Surgery, BG Trauma Center Murnau, Murnau am Staffelsee, Germany

**Keywords:** Awareness of predatory journals, Orthopaedic and trauma surgeons, Online survey, Open access journals

## Abstract

**Background:**

Along with emerging open access journals (OAJ) predatory journals increasingly appear. As they harm accurate and good scientific research, we aimed to examine the awareness of predatory journals and open access publishing among orthopaedic and trauma surgeons.

**Methods:**

In an online survey between August and December 2019 the knowledge on predatory journals and OAJ was tested with a hyperlink made available to the participants via the German Society for Orthopaedics and Trauma Surgery (DGOU) email distributor.

**Results:**

Three hundred fifty orthopaedic and trauma surgeons participated, of which 291 complete responses (231 males (79.4%), 54 females (18.6%) and 5 N/A (2.0%)) were obtained. 39.9% were aware of predatory journals. However, 21.0% knew about the “Directory of Open Access Journals” (DOAJ) as a register for non-predatory open access journals. The level of profession (e.g. clinic director, consultant) (*p* = 0.018) influenced the awareness of predatory journals. Interestingly, participants aware of predatory journals had more often been listed as corresponding authors (*p* < 0.001) and were well published as first or last author (*p* < 0.001). Awareness of OAJ was masked when journal selection options did not to provide any information on the editorial board, the peer review process or the publication costs.

**Conclusion:**

The impending hazard of predatory journals is unknown to many orthopaedic and trauma surgeons. Early stage clinical researchers must be trained to differentiate between predatory and scientifically accurate journals.

**Supplementary Information:**

The online version contains supplementary material available at 10.1186/s12891-021-04223-7.

## Introduction

Spreading newly found knowledge is as important as acquiring knowledge itself. Beside writing monographs and presenting research results on symposia and conferences, writing papers and publishing them in scientific journals is a long-established method to inform the scientific community about recent advances in different research fields. In orthopaedic and trauma surgery, peer-review journals have been established at the turn of the nineteenth to twentieth century. Since then, it was common practice to disseminate knowledge in journals as printed media [1]. With the internet revolution in the late twentieth century the business model of scholarly publishing experienced a transition from selling printed format journals to new online business models such as selling either single articles, subscriptions or an author charging model called “open access” (OA) [2]. This publishing model gets funded by article processing charges (APCs) providing freely available articles all over the world, which is very beneficial for interested readers who cannot afford paid subscriptions or have no institutional access to scientific journals. However, the upside of freely accessible scientific literature through fee-charging journal incites predatory journals, which pose a major threat to academics. Predatory journals are fake or trash journals and exist for the sole purpose to make profit. The journals only look like genuine scientific journals and try to mislead inexperienced scientists. They pretend to achieve a fast peer-review process and promise fast publication of manuscripts. Beall defines predatory OA publishers by arguing that their primary goal is to deceive both authors and readers through a lack of transparency in their internal processes. He claims these publishers charge a fee but do not provide the expected and advertised services [3]. Some of them use journal names similar to well-recognized journals pretending authenticity [4] and often claim to be indexed in the main scientific databases such as PubMed, Web of Science or Scopus. To pretend their notoriety, adjectives such as “world” or “global” are included in the title [3]. Besides, the falsely acclaimed impact factor on predatory journals websites is deluding [5]. Predatory journals also ignore quality standards, such as the scientific content of the paper, disclosure of conflict of interests and handling misprints, which are recommended by associations such as WAME (World Association of Medical Editors), the International Committee of Medical Journal Editors (ICMJE), the Committee on Publication Ethics (COPE), and the Council of Science Editors (CSE) [6]. Furthermore, publishing articles without peer-review process as quality control provides questionable science with false merit [5, 7]. It is generally assumed, that this dubious practice threatens especially young academics who are in need to publish and not perish in the academic arena [8]. In the orthopaedic field, data about the awareness of predatory journals is missing. Thus, we aimed to investigate the knowledge of orthopaedic and trauma surgeons about this important topic.

## Participants and methods

This study is based on an online survey, with a hyperlink made available to the participants via the German Society for Orthopaedics and Trauma Surgery (DGOU) email distributor. The DGOU is a medical-scientific professional society founded in 2008 as a non-profit association. Its responsibilities include representing the interests of research, teaching, postgraduate education and training in the field of orthopaedics and trauma surgery. Orthopaedic and trauma surgeons irrespective of their educational level or involvement in research, but also students engaged in orthopaedic and trauma surgery as well as retired orthopaedic and trauma surgeons were eligible to participate. The professional expertise was determined according to the categories: student, resident, junior consultant, senior consultant, clinic director, private practitioner, retired / various. Access to the electronic questionnaire on the DGOU homepage via the hyperlink was possible from August through December 2019.

The survey was divided into five parts. Within the first section, participants were asked to provide general information about themselves (age, gender, professional and academic status). The second part covered the latest employer and the field of professional activity. The third section dealt with the type of research at the institution and the number of papers already published. Section four investigated the level of awareness of “predatory journals” and pseudo-scientific research. Whereby the knowledge on predatory journals was captured with a yes-no decision question (“Have you ever heard of Predatory Journals?”). The question about knowledge of the JCR list, contained supplementary the term Thomson Reuters list in our survey, as the terms frequently appear combined. The last section focused on open access publishing, using single-choice questions with a 5-point Likert scale, reaching from “strongly agree” to “strongly disagree”, plus a check box for “not specified”.

### Ethical agreement

Ethical approval and informed consent has been waived off by the responsible Institutional Review Board (IRB) of the University Medical Center Regensburg. No personal data (including the IP address) were collected. This study does not include experiments on humans or animals. It is completely limited to the collection of data regarding the publication process. The study has been conducted in accordance with the Declaration of Helsinki as revised in 2013.

### Statistics

The electronic data collection was performed with the web-based survey tool for business purposes, established by the company “Survey Monkey” (https://de.surveymonkey.com/). The survey was anonymized, and it was ensured that tracing of participants via IP address was not possible. Extracted data was then transformed into an Excel file and imported to SPSS (IBM SPSS Statistics 25) for statistical analysis. The data was analyzed using the Chi-square test. In case that more than 20% of the cells had a cell frequency lower than five, the Fisher-Yates test was used. In order to highlight the association between the scientific publications and the professional position as well as the received e-mail requests, the findings were color-coded in Excel according to the percentage coherence. The significance level was set at an alpha of 5%.

## Results

A total of 350 members of the DGOU participated in this online survey. Fifty-eight incomplete datasets had to be excluded. Thus, 291 individual responses were analyzed representing a response rate of 4.0%.

**Fig. 1:**
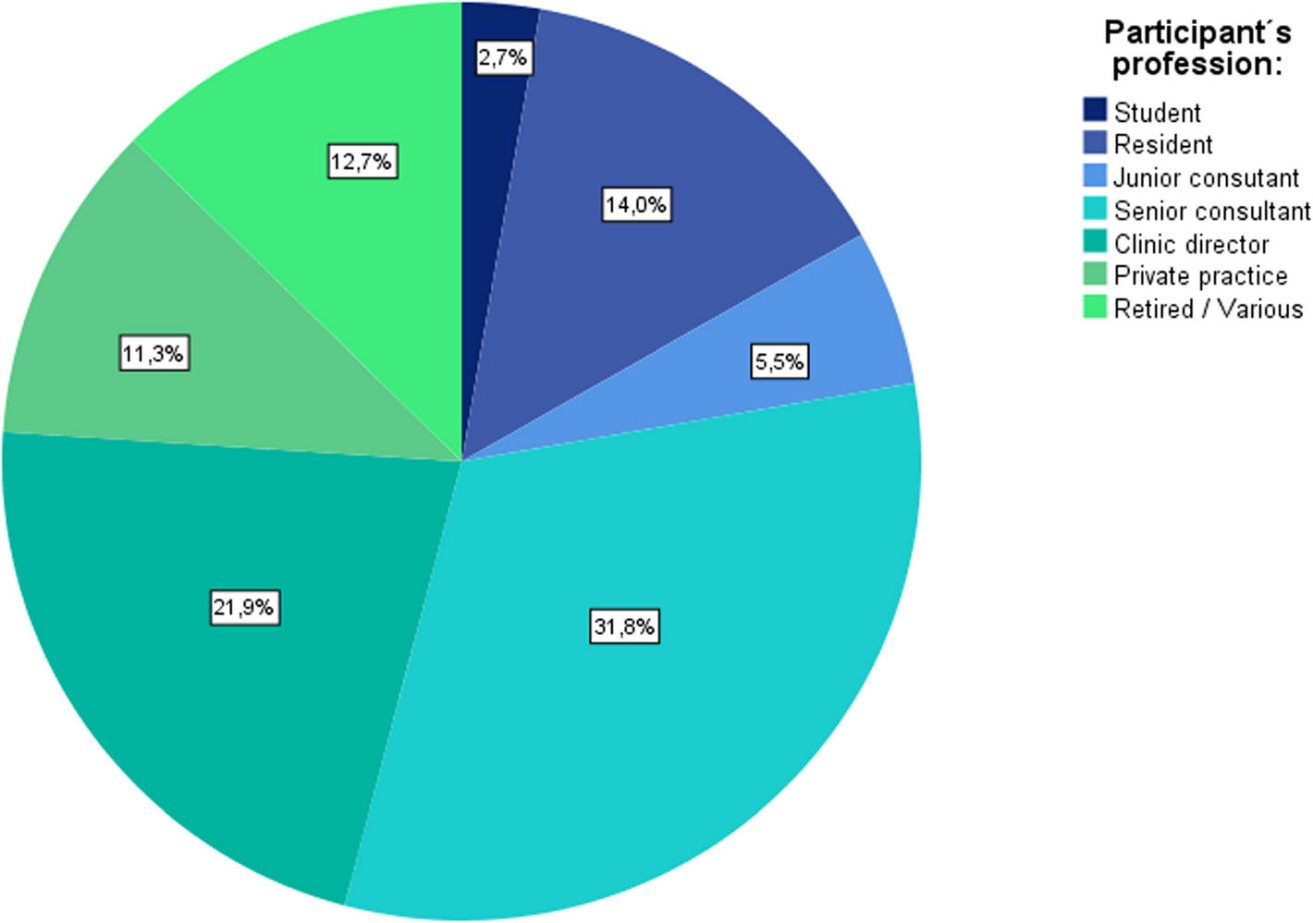
Participant’s profession

Two hundred thirty-one males (79.4%), 54 females (18.6%) and 6N/A (2.0%) completed the survey. 78.4% (*n* = 228) of the participants were aged between 30 and 60 years. 93 (31.8%) senior consultants, 64 (21.9%) clinic directors, 41 (14.0%) residents, 33 (11.3%) private practice-based orthopaedic and trauma surgeons (PPOT), 16 (5.5%) junior consultants and eight students (2.7%) took part in the survey. Further, 37 (12.7%) were retired or did not belong to any of the above-mentioned groups (Fig. 1).

One hundred sixteen participants (39.9%) were aware of predatory journals. Neither age (*p* = 0.558), nor gender (*p* = 0.768), nor the field of professional activity (*p* = 0.163) significantly influenced the knowledge on predatory journals. In contrast, professional occupation (*p* = 0.018), but also the workplace (*p* < 0.001) significantly correlated with knowledge on predatory journals. A detailed description of the demographic characteristics, employment / workplace and field of professional activity is given in Table 1. Here, a distinction was made in each case according to knowledge of predatory journals.

**Table 1:** A detailed description of the demographic characteristics, employment and field of professional activity divided by the knowledge on predatory journals

	All participants *N* = 291	Knowledge of predatory journals		
		Yes *N* = 116	No *N* = 175	***p***-value
Sex, n (%)				
Male	231 (79,4)	95 (81,9)	136 (77,7)	0,768
Female	54 (18,6)	19 (16,4)	35 (20,0)	
N / A	6 (2,0)	2 (1,7)	4 (2,3)	
Age, years (± SD)				
< 30	14 (4,8)	8 (6,9)	6 (3,4)	0,558
30–40	79 (27,1)	31 (26,7)	48 (27,4)	
41–50	67 (23,0)	26 (22,4)	41 (23,4)	
51–60	82 (28,2)	29 (25,0)	53 (30,3)	
> 60	49 (16,8)	22 (19,0)	27 (15,4)	
Professional occupation, n (%)				
Student	8 (2,7)	6 (5,2)	2 (1,1)	**0,018**
Resident	41 (14,1)	13 (11,2)	28 (16,0)	
Junior consultant	16 (5,5)	6 (5,2)	10 (5,7)	
Senior consultant	93 (32,0)	34 (29,3)	59 (33,7)	
Clinic director	63 (21,6)	27 (23,3)	36 (20,6)	
PPOT^a^	33 (11,3)	8 (6,9)	25 (14,3)	
Retired / Various	37 (12,7)	22 (19,6)	15 (8,6)	
Passed promotion, n (%)				
Yes	243 (83,5)	103 (88,8)	140 (80,0)	**0,048**
No	48 (16,5)	13 (11,2)	35 (20,0)	
Passed habilitation, n (%)				
Yes	101 (34,7)	62 (53,4)	39 (22,3)	**< 0,001**
No	190 (65,3)	54 (46,6)	136 (77,7)	
Passed professorship, n (%)				
Yes	62 (21,3)	44 (37,9)	18 (10,3)	**< 0,001**
No	229 (78,7)	72 (62,1)	157 (89,7)	
Employer / Workplace, n (%)				
Basic and standard care hospital	51 (17,5)	7 (6,0)	44 (25,1)	**< 0,001**
University Hospital	71 (24,4)	45 (38,8)	26 (14,9)	
Teaching hospital	95 (32,5)	31 (26,7)	63 (36,1)	
Rehab Clinic	8 (2,7)	4 (3,4)	4 (2,3)	
Private practice	31 (10,7)	10 (8,6)	21 (12,0)	
Research / Laboratory	18 (6,2)	15 (12,9)	3 (1,7)	
Various	18 (6,2)	4 (3,4)	14 (8,0)	
Field of professional activity, n (%)				
Orthopaedics	60 (20,6)	29 (25,0)	31 (17,7)	0,163
Trauma surgery	66 (22,7)	30 (25,9)	36 (20,6)	
Orthopaedics & trauma surgery	140 (48,1)	50 (43,1)	90 (51,4)	
Various	25 (8,6)	7 (6,0)	18 (10,3)	

^a^*PPOT* private practice-based orthopaedics and/or trauma surgeons

Only 29.6% of the participants were aware of the “think, check and submit” approach for publishing (*p* < 0.001), and even less (21.0%) have ever heard of the “Directory of Open Access Journals” (DOAJ) (*p* < 0.001). In addition, participants with knowledge on predatory journals had more often been listed as corresponding authors (*p* < 0.001) and had a higher number of published papers as first or last author (*p* < 0.001) (Table 2).

**Table 2:** A detailed description of the number of publications and open access publishing, depending on the knowledge of predatory journals

	All participants *N* = 291	Knowledge of predatory journals		
		Yes *N* = 116	No *N* = 175	***p***-value
Is there any possibility to do research at your work place, n (%)				
Yes	227 (78,0)	108 (93,1)	119 (68,0)	**< 0,001**
No	64 (22,0)	8 (6,9)	56 (32,0)	
Total number of publications, n (%)				
0	48 (16,5)	13 (11,2)	35 (26,0)	**< 0,001**
1	31 (10,7)	5 (4,3)	26 (14,9)	
2–5	55 (18,9)	7 (6,0)	48 (27,4)	
6–10	23 (7,9)	9 (7,8)	14 (8,0)	
11–20	33 (11,3)	15 (12,9)	18 (10,3)	
> 21	101 (34,7)	67 (57,8)	34 (14,4)	
Number of publications (first/ last author), n (%)				
0	63 (21,6)	15 (12,9)	48 (27,4)	**< 0,001**
1	37 (12,7)	5 (4,3)	32 (18,3)	
2–5	66 (22,7)	21 (18,1)	45 (35,7)	
6–10	25 (8,6)	9 (7,8)	16 (9,1)	
11–20	33 (11,3)	20 (17,2)	13 (7,4)	
> 21	67 (23,1)	46 (39,7)	21 (12,1)	
Have you published anything in an open access journal (OA), n (%)				
Yes	118 (40,5)	74 (63,8)	44 (25,1)	**< 0,001**
No	173 (59,5)	42 (36,2)	131 (74,9)	
Have you heard of the “Directory of Open Access Journals” (DOAJ), n (%)				
Yes	61 (21,0)	40 (34,5)	21 (12,0)	**< 0,001**
No	230 (79,0)	76 (65,5)	154 (88,0)	
Are you familiar with the Think, Check and Submit approach before publishing, n (%)				
Yes	86 (29,6)	72 (62,1)	14 (8,0)	**< 0,001**
No	205 (70,4)	44 (37,9)	161 (92,0)	

Two hundred forty-three participants (93.5%) have published at least one paper, either as co-author, first or last author. 118 (40.5%) have published a manuscript in an OA journal. The total number of publications (*p* < 0.05) and the number of first or last authorships (*p* < 0.001) were significantly correlated with the professional occupation (Table 2).

The number of papers published showed a strong positive correlation (*r* = 0.742, *p* < 0.001) with the number of e-mail requests received per week. 10.5% of those participants who did not report any publication, stated to receive at least one invitation by e-mail. The probability of receiving advertising e-mails increases with the number of publications (Detailed data is attached in the supplements).

**Fig. 2:**
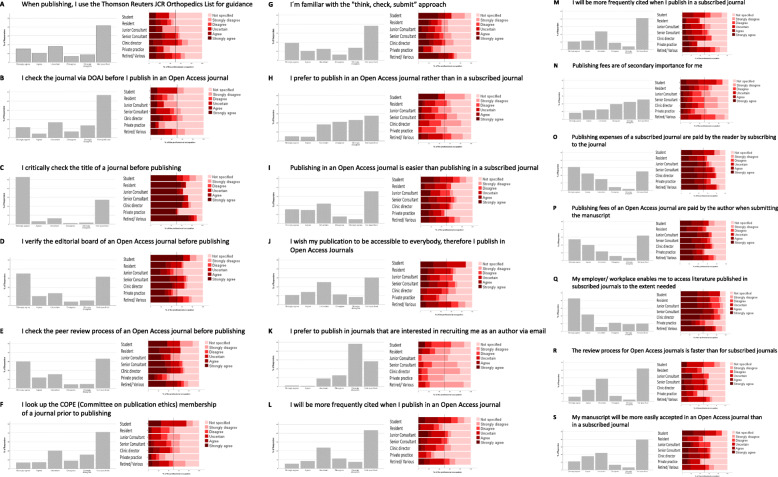
**a**-**g** Own approach to publishing. Display of the distribution of the answers. Left: Overall evaluation without grouping. Right: Subdivision according to the professional expertise. **h**, **j**, **k**, **n** Preferences in Publishing. **i**, **l**, **m**, **s**, **r** Expectations, **q** access to literature and **o** and **p** knowledge on publication fees

The survey records the level of knowledge in various areas. First, the own approach to publishing was assessed (Fig. 2a-g). In response to the question whether one should critically review the journal before submitting it, most participants answered “strongly agree”. Whether a review of the Journal Citation Record (JCR) list or the DOAJ is performed was answered with “not specified” by the majority of participants. Second, publishing preferences were surveyed and evaluated (Fig. 2h, j, k, n). Regarding the preferred publication in an Open Access journal instead of a conventional journal, the survey showed a clear trend towards rejection (“uncertain”, “disagree”, “strongly disagree”). Following this, the expectations in relation to the journal and the citation were evaluated (Fig. 2i, l, m, s, r). While the majority of the respondents stated that the required literature was provided by the employer / workplace or via online access (Fig. 2q), there was no consensus on the review process of an Open Access journal. On the one hand, there was a trend of opinion that it is easier and faster to publish in an Open Access journal. On the other hand, a larger proportion of those questioned did not define the procedure (“not specified”). However, there was agreement on a) whether the publication costs of a subscription journal are covered by the reader and b) whether the publication fees of an Open Access journal are paid by the publisher (Fig. 2O AND P).

## Discussion

To the best of our knowledge, this is the first online survey in the field of orthopaedic and trauma surgery to specifically address the current awareness of predatory journals and open access publishing in Germany. We found that only 39.9% of all participants were aware of predatory journals. Age, gender or the field of professional activity did not correlate with the knowledge on predatory journals. Regardless of the medical subspecialty, knowledge about predatory journals is alarmingly limited. In line with our findings, the prevalence of awareness varies between 29.4% in dermatology and 69.7% in oncology [9, 10]. In this concern, Cohen et al. interviewed several editorial publishers who are listed as editors of a predatory journal, showing that 77.0% reported a high level of knowledge on the danger associated with predatory journals. However, 40.0% of the contacted editors were not aware of being an editor for this journal in particular [11].

Our data demonstrated, that both the professional occupation and the employer / working place impacts the knowledge of predatory journals. Surprisingly, the knowledge of the underlying danger was very high, particularly among students. However, this could be based on a bias, as this survey only included individual students (*n* = 8) who had made early contributions in the field of orthopaedics and trauma surgery, for example through scientific lectures on a congress. Furthermore, a more fundamental comparison with the literature is not possible due to a lack of surveys among students, but would be very informative both before and after the integrated academic course on “good clinical practice”.

Only 31.7% of the residents knew the term “predatory journals”. In comparison knowledge in dermatological residents was lower (20.3%) [9] whilst awareness in oncological residents (47.8%) [10] was much more pronounced. Whereas the awareness level of the predatory journals among junior consultants (37.5%) and senior consultants (36.6%) within our collective was similar to that of the residents, the familiarity of the predatory journals in dermatology showed an increase to 33.3% among senior consultants. However, the share in oncology consultants remained similar to that of residents at 46.3% [9, 10]. Furthermore, there was a close association between the level of scientific expertise and the knowledge of predatory journals. This is reflected by the fact that 71% of the professors and 61.4% of the colleagues who achieved the “habilitation”, which is a qualification required in order to conduct self-contained university teaching, were familiar with the issue. This is in line with the findings by Richtig et al. [9]. Christopher et al. emphasized that the term predatory journals is often misunderstood and the danger is thus further underestimated. As especially trainees were unaware of the hazard, they recommend early education of younger professionals, [12]. As previously described in the literature, our findings also indicate that participants employed by university hospitals or research institutions were substantially more likely to be aware of the threat posed by predatory journals [9, 10].

Nevertheless, knowledge about Open Access journals seems to be very limited. Although the majority of the surveyed had a clear understanding of how to cover the costs of a conventional or an Open Access journal, the ideas about the publishing process differed considerably. This becomes clear, as for example a relatively large proportion of participants answered questions about the editorial board and the peer review process with “not specified”. To the opposite, 63.6% of the respondents stated that they would critically review the title of the journal before submission and 45.4% claimed to critically review the editorial board. Surprisingly low was the proportion (6.3%) of those who answered the question “I prefer to publish in an Open Access journal rather than in a subscription journal” with strongly agree / agree. 31.3% were convinced that it was easier to publish in an open access journal than in a conventional journal. This study did not investigate the impact factor’s influence on the planned publication, but the literature clearly showed that this was a decisive factor for many authors [13]. Furthermore, publication fees are an important factor in the decision-making process when considering publishing. Koroulakis et al. showed that more than 50% of the residents made their choice to publish at least once dependent on publication fees [13]. In our cohort, only 19.9% of participants stated that publication costs play a secondary role. 12.7% were uncertain, while for 41.9% the publication costs were a decisive factor.

29.6% of all participants were familiar with the “think, check and submit” approach, while less (21.0%) have ever heard about the DOAJ. In contrast, Swanberg et al. showed that almost 50% of the study participants knew that journals listed in the DOAJ were considered legitimate [14]. Therefore, it is important, that already alongside their academic education, young academics are side advised of the possible dangers by predatory journals on the one hand and on the other hand learn about the “think, check and submit” approach, the Journal Citation Reports (JCR) list and the DOAJ with mandatory courses on “good clinical practice”. Since even a co-authorship in a predatory journal can lead to great harm, the small effort to identify fake journals before submission should be made [15]. While the effects may seem limited at first, we think it might become apparent in the longer term, for example through incorrect treatment recommendations in patient care. Only a sincere peer review process can help to avoid misleading literature.

Richtig et al. reported that three quarters of all interviewed participants regularly received advertising e-mails with requests/offers for publication from different journals. In most cases (77.5%) these were from predatory journals [10]. We found, that the number of papers published positively correlated with the number of e-mail requests received per week and with the professional occupation. However, this does not only affect the co-responding author, whose e-mail contact is easily available on the published paper. The question is, how can publishers protect authors from these unpleasant daily annoyances? Are contact details only to be given out on justified request? Recently, this problem is gaining increased attention.

Although the study reveal a bigger threat of predatory journals as before thought, some limitations governed the resulting conclusion. The main limitation of this study is that we only surveyed orthopaedists and trauma surgeons who were included in the mailing list of the German Society for Orthopaedics and Trauma Surgery (DGOU) online. Thus, not all orthopaedic and trauma surgeons in Germany could be reached. Second, the overall response rate of 4% was low. Nevertheless, our results are similar to those described in the literature. Perhaps in a shortened questionnaire more than 291 of the 350 participants would have answered all questions, leading to a smaller drop-out rate.

Overall, we consider our results to be representative for Germany. We base it on the fact that, according to the Federal Medical Association (Bundesärztekammer), 19.158 orthopaedic and trauma surgeons are registered in Germany. About half of them are members of the DGOU. Thus, our survey was made available to almost 50% of the orthopaedic and trauma surgeons in Germany. According to the DGOU, 10,2% of members are clinic directors, 19,9% are senior consultants, and 16.7% are junior consultants. Our response rate deviates slightly from this to an excess representation of clinic directors and senior consultants. However, this deviation is negligible from our point of view.

## Conclusion

The impending hazard of predatory journals is unknown to many orthopaedic and trauma surgeons. The present results underline the need to improve awareness of predatory journals among young colleagues to avoid unintentional scientific missteps and lasting career damage. For this purpose, courses about scientific publishing as part of medical school or later during residency programs seem useful to guide and protect young colleagues from the beginning of their professional career.

## Supplementary Information

**Additional file 1: Supplement 1.** Relation between the number of papers published and the number of e-mail requests received per week. **Supplement 2**. Results of the survey as a function of knowledge about Predatory Journals

## Data Availability

The data that support the findings of this study are available on reasonable request from the corresponding author.
