# Supplementary material for: Awareness of predatory journals and open access publishing among orthopaedic and trauma surgeons – results from an online survey in Germany
Source: BMC Musculoskelet Disord. 2021 Apr 17;22:365. doi: 10.1186/s12891-021-04223-7 (PMC8053264; doi:10.1186/s12891-021-04223-7)

**Supplement 1**. Relation between the number of papers published and the number of e-mail requests received per week.


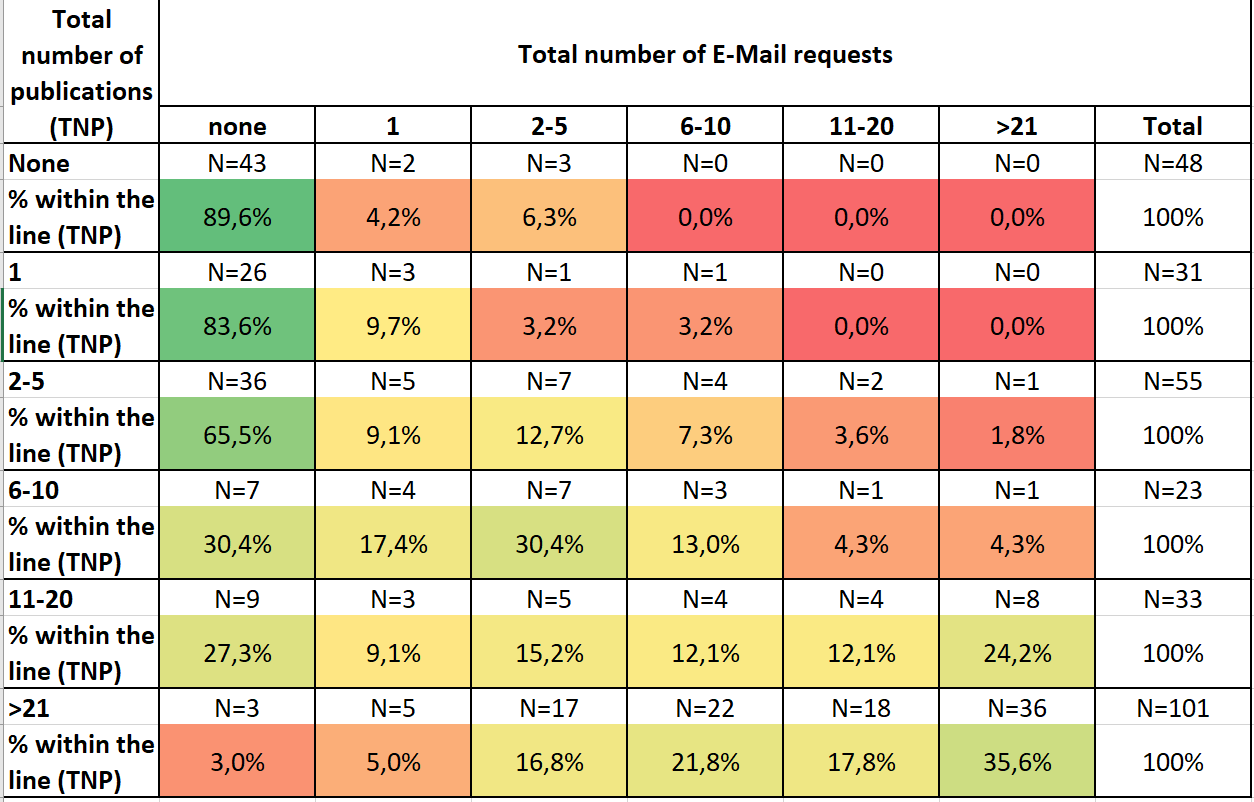

Supplement: Supplementary file 1 — Additional file 1: Supplement 1. Relation between the number of papers published and the number of e-mail requests received per week. Supplement 2. Results of the survey as a function of knowledge about Predatory Journals [file 12891_2021_4223_MOESM1_ESM.zip › supplements/Supplement 1.docx]
