# Supplementary material for: Awareness of predatory journals and open access publishing among orthopaedic and trauma surgeons – results from an online survey in Germany
Source: BMC Musculoskelet Disord. 2021 Apr 17;22:365. doi: 10.1186/s12891-021-04223-7 (PMC8053264; doi:10.1186/s12891-021-04223-7)
Supplement: Supplementary file 1 — Additional file 1: Supplement 1. Relation between the number of papers published and the number of e-mail requests received per week. Supplement 2. Results of the survey as a function of knowledge about Predatory Journals [file 12891_2021_4223_MOESM1_ESM.zip › supplements/Supplement 2.docx]

**Supplement 2**. Results of the survey as a function of knowledge about Predatory Journals

|  |  | **Knowledge of predatory journals** | | |
| --- | --- | --- | --- | --- |
|  | **All participants**  N=291, n  (% in column) | **Yes**  N=116, n (% in line) | **No**  N=175, n  (% in line) | **p-value** |
| When publishing, I use the Thomson Reuters JCR Orthopedics List for guidance, n (% in line)  Strongly agree  Agree  Uncertain  Disagree  Strongly disagree  Not specified | 44 (15,1)  30 (10,3)  52 (17,9)  19 (6,5)  25 (8,6)  121 (41,6) | 29 (25,0)  21 (18,1)  26 (22,4)  7 (6,0)  9 (7,8)  24 (20,7) | 15 (8,6)  9 (5,1)  26 (14,9)  12 (6,9)  16 (9,1)  97 (55,4) | **<0,001**  **Chi** |
| I check the journal via DOAJ before I publish in an Open Access journal, n (% in line)  Strongly agree  Agree  Uncertain  Disagree  Strongly disagree  Not specified | 34 (11,7)  13 (4,5)  49 (16,8)  20 (6,9)  39 (13,4)  136 (46,7) | 24 (20,7)  8 (6,9)  26 (22,4)  11 (9,5)  19 (16,4)  28 (24,1) | 10 (5,7)  5 (2,9)  23 (13,1)  9 (5,1)  20 (11,4)  108 (61,7) | **<0,001**  **Chi** |
| I critically check the title of a journal before publishing, n (% in line)  Strongly agree  Agree  Uncertain  Disagree  Strongly disagree  Not specified | 176 (60,5)  9 (3,1)  19 (6,5)  3 (1,0)  5 (1,7)  79 (27,1) | 97 (83,6)  4 (3,4)  6 (5,2)  1 (0,9)  1 (0,9)  7 (6,0) | 79 (45,1)  5 (2,9)  13 (7,4)  2 (1,1)  4 (2,3)  72 (41,1) | **<0,001**  **Fisher** |
| I verify the editorial board of an Open Access journal before publishing, n (% in line)  Strongly agree  Agree  Uncertain  Disagree  Strongly disagree  Not specified | 102 (35,1)  30 (10,3)  39 (13,4)  12 (4,1)  16 (5,5)  92 (31,6) | 62 (53,4)  19 (16,4)  13 (11,2)  5 (4,3)  7 (6,0)  10 (8,6) | 40 (22,9)  11 (6,3)  26 (14,9)  7 (4,0)  9 (5,1)  82 (46,9) | **<0,001**  **Chi** |
| I check the peer review process of an Open Access journal before publishing, n (% in line)  Strongly agree  Agree  Uncertain  Disagree  Strongly disagree  Not specified | 87 (29,9)  35 (12,0)  46 (15,8)  13 (4,5)  14 (4,8)  96 (33,0) | 52 (44,8)  23 (19,8)  19 (16,4)  4 (3,4)  7 (6,0)  11 (9,5) | 35 (20,0)  12 (6,9)  27 (15,4)  9 (5,1)  7 (4,0)  85 (48,6) | **<0,001**  **Chi** |
| I look up the COPE (Committee on publication ethics) membership of a journal prior to publishing, n (% in line)  Strongly agree  Agree  Uncertain  Disagree  Strongly disagree  Not specified | 20 (6,9)  21 (7,2)  59 (20,3)  26 (8,9)  46 (15,8)  119 (40,9) | 14 (12,1)  16 (13,8)  33 (28,4)  13 (11,2)  21 (18,1)  19 (16,4) | 6 (3,4)  5 (2,9)  26 (14,9)  13 (7,4)  25 (14,3)  100 (57,1) | **<0,001**  **Chi** |
| I´m familiar with the "think, check, submit” approach, n (% in line)  Strongly agree  Agree  Uncertain  Disagree  Strongly disagree  Not specified | 56 (19,2)  30 (10,3)  36 (12,4)  18 (6,2)  40 (13,7)  111 (38,1) | 39 (33,6)  22 (19,0)  17 (14,7)  7 (6,0)  11 (9,5)  20 (17,2) | 17 (9,7)  8 (4,6)  19 (10,9)  11 (6,3)  29 (16,6)  91 (52,0) | **<0,001**  **Chi** |
| I prefer to publish in an Open Access journal rather than in a subscribed journal, n (% in line)  Strongly agree  Agree  Uncertain  Disagree  Strongly disagree  Not specified | 7 (4,0)  4 (2,3)  21 (12,0)  36 (20,6)  33 (18,9)  74 (42,3) | 8 (6,9)  10 (8,6)  32 (27,6)  25 (21,6)  34 (29,3)  7 (6,0) | 15 (5,2)  14 (4,8)  53 (18,2)  61 (21,0)  67 (23,0)  81 (27,8) | **<0,001**  **Chi** |
| Publishing in an Open Access journal is easier than publishing in a subscribed journal, n (% in line)  Strongly agree  Agree  Uncertain  Disagree  Strongly disagree  Not specified | 45 (15,5)  43 (14,8)  64 (22,0)  22 (7,6)  13 (4,5)  104 (35,7) | 20 (17,2)  25 (21,6)  28 (24,1)  14 (12,1)  13 (11,2)  16 (13,8) | 25 (14,3)  18 (10,3)  36 (20,6)  8 (4,6)  0 (0,0)  88 (50,3) | **<0,001**  **Chi** |
| I wish my publication to be accessible to everybody, therefore I publish in Open Access Journals, n (% in line)  Strongly agree  Agree  Uncertain  Disagree  Strongly disagree  Not specified | 31 (10,7)  41 (14,1)  73 (25,1)  33 (11,3)  25 (8,6)  88 (30,2) | 17 (14,7)  28 (24,1)  32 (27,6)  13 (11,2)  13 (11,2)  13 (11,2) | 14 (8,0)  13 (7,4)  41 (23,4)  20 (11,4)  12 (6,9)  75 (42,9) | **<0,001**  **Chi** |
| I prefer to publish in journals that are interested in recruiting me as an author via email, n (% in line)  Strongly agree  Agree  Uncertain  Disagree  Strongly disagree  Not specified | 3 (1,0)  2 (0,7)  26 (8,9)  37 (12,7)  140 (48,1)  83 (28,5) | 2 (1,7)  0 (0,0)  5 (4,3)  20 (17,2)  80 (69,0)  9 (7,8) | 1 (0,6)  2 (1,1)  21 (12,0)  17 (9,7)  60 (34,3)  74 (42,3) | **<0,001**  **Fisher** |
| I will be more frequently cited when I publish in an Open Access journal, n (% in line)  Strongly agree  Agree  Uncertain  Disagree  Strongly disagree  Not specified | 16 (5,5)  25 (8,6)  69 (23,7)  33 (11,3)  22 (7,6)  126 (43,3) | 10 (8,6)  19 (16,4)  38 (32,8)  17 (14,7)  12 (10,3)  20 (17,2) | 6 (3,4)  6 (3,4)  31 (17,7)  16 (9,1)  10 (5,7)  106 (60,6) | **<0,001**  **Chi** |
| I will be more frequently cited when I publish in a subscribed journal, n (% in line)  Strongly agree  Agree  Uncertain  Disagree  Strongly disagree  Not specified | 31 (10,7)  34 (11,7)  69 (23,7)  26 (8,9)  12 (4,1)  119 (40,9) | 13 (11,2)  18 (15,5)  42 (36,2)  15 (12,9)  9 (7,8)  19 (16,4) | 18 (10,3)  16 (9,1)  27 (15,4)  11 (6,3)  3 (1,7)  100 (57,1) | **<0,001**  **Chi** |
| Publishing fees are of secondary importance for me, n (% in line)  Strongly agree  Agree  Uncertain  Disagree  Strongly disagree  Not specified | 24 (8,2)  34 (11,7)  37 (12,7)  57 (19,6)  65 (22,3)  74 (25,4) | 12 (10,3)  20 (17,2)  23 (19,8)  27 (23,3)  26 (22,4)  8 (6,9) | 12 (6,9)  14 (8,0)  14 (8,0)  30 (17,1)  39 (22,3)  66 (37,7) | **<0,001**  **Chi** |
| Publishing expenses of a subscribed journal are paid by the reader by subscribing to the journal, n (% in line)  Strongly agree  Agree  Uncertain  Disagree  Strongly disagree  Not specified | 90 (30,9)  65 (22,3)  44 (15,1)  13 (4,5)  6 (2,1)  73 (25,1) | 40 (34,5)  36 (31,0)  18 (15,5)  6 (5,2)  6 (5,2)  10 (8,6) | 50 (28,6)  29 (16,6)  26 (14,8)  7 (4,0)  0 (0,0)  63 (36,0) | **<0,001**  **Chi** |
| Publishing fees of an Open Access journal are paid by the author when submitting the manuscript, n (% in line)  Strongly agree  Agree  Uncertain  Disagree  Strongly disagree  Not specified | 79 (27,1)  58 (19,9)  34 (11,7)  17 (5,8)  10 (3,4)  93 (32,0) | 46 (39,7)  35 (30,2)  14 (12,1)  4 (3,4)  5 (4,3)  12 (10,3) | 33 (18,9)  23 (13,1)  20 (11,4)  13 (7,4)  5 (2,9)  81 (46,3) | **<0,001** |
| My employer/ workplace enables me to access literature published in subscribed journals to the extent needed, n (% in line)  Strongly agree  Agree  Uncertain  Disagree  Strongly disagree  Not specified | 124 (42,6)  63 (21,6)  16 (5,5)  32 (11,0)  27 (9,3)  29 (10,0) | 58 (50,0)  30 (25,9)  5 (4,3)  12 (10,3)  7 (6,0)  4 (3,4) | 66 (37,7)  33 (18,9)  11 (6,3)  20 (11,4)  20 (11,4)  25 (14,3) | **<0,05**  **Chi** |
| The review process for Open Access journals is faster than for subscribed journals, n (% in line)  Strongly agree  Agree  Uncertain  Disagree  Strongly disagree  Not specified | 17 (5,8)  42 (14,4)  84 (28,9)  20 (6,9)  6 (2,1)  122 (41,9) | 9 (7,8)  22 (19,0)  51 (44,0)  10 (8,6)  5 (4,3)  19 (16,4) | 8 (4,6)  20 (11,4)  33 (18,9)  10 (5,7)  1 (0,6)  103 (58,9) | **<0,001**  **Chi** |
| My manuscript will be more easily accepted in an Open Access journal than in a subscribed journal, n (% in line)  Strongly agree  Agree  Uncertain  Disagree  Strongly disagree  Not specified | 32 (11,0)  52 (17,9)  64 (22,0)  19 (6,5)  9 (3,1)  115 (39,5) | 12 (10,3)  31 (26,7)  30 (25,9)  14 (12,1)  8 (6,9)  21 (18,1) | 20 (11,4)  21 (12,0)  34 (19,4)  5 (2,9)  1 (0,6)  94 (53,7) | **<0,001**  **Chi** |
